# Ultraviolet exposure has an epigenetic effect on a Batesian mimetic trait in the butterfly *Papilio polytes*

**DOI:** 10.1038/s41598-018-31732-8

**Published:** 2018-09-07

**Authors:** Mitsuho Katoh, Haruki Tatsuta, Kazuki Tsuji

**Affiliations:** 1 Department of Agro-Environmental Sciences, Faculty of Agriculture, University of the Ryukyus, Okinawa, 903-0213 Japan; 2 The United Graduate School of Agricultural Sciences, Kagoshima University, Korimoto 1-21-24, Kagoshima, 890-8580 Japan

## Abstract

Wing polymorphism of butterflies provides a good system in which to study adaptation. The Asian Batesian mimic butterfly *Papilio polytes* has unmelanized, putative mimetic red spots on its black hind wings. The size of those red spots is non-heritable but it is highly polymorphic, the adaptive significance of which is unknown. We hypothesized that under strong ultraviolet (UV) irradiation, butterflies develop a wider melanized black area to protect the wings from UV damage, and as a result express smaller mimetic red spots. Our field survey on Okinawa Island revealed a negative relationship between the sizes of the red spot and the black area in the wings. The size varied seasonally and was negatively correlated with the intensity of solar UV radiation at the time of capture. Laboratory experiments revealed that the size was reduced by strong UV irradiation not only of the eggs and larvae, but also of their mothers through a putative epigenetic mechanism. The flexible phenotypic expression of the red spots in *P*. *polytes* suggests a trade-off between protection against UV damage and predation avoidance, and provides a new insight into the evolution of Batesian mimicry.

## Introduction

**Figure 1:**
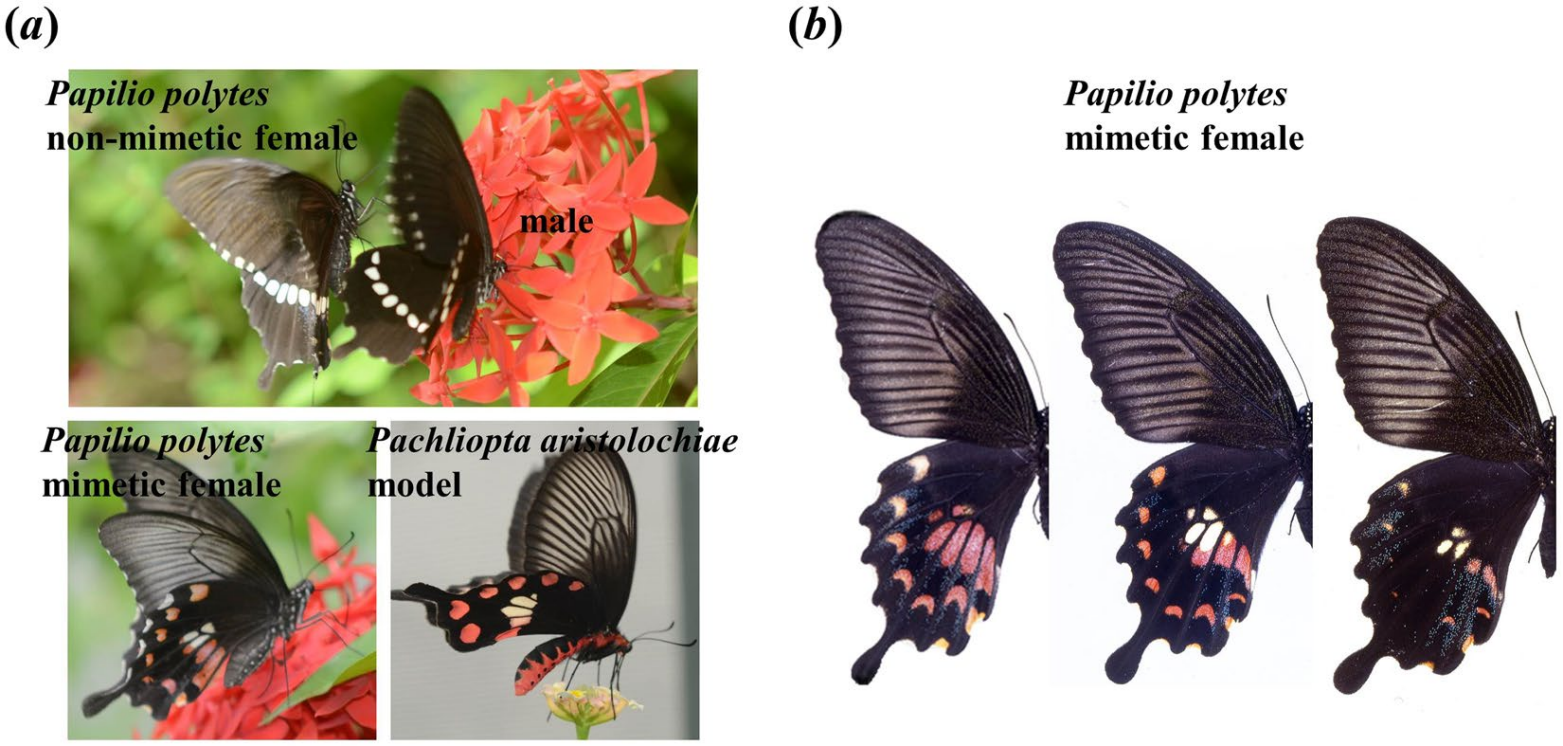
*Papilio polytes* and its noxious model. (**a**) Top: *Papilio polytes* f. *cyrus* non-mimetic female (left) and male (right); lower-left: f. *polytes* mimetic female; lower-right: the poisonous model, *Pachliopta aristolochiae*. (**b**) The red spot size of f. *polytes* on Okinawa varies widely. All specimens were collected by the authors.

Wing morphology of butterflies is considered a strongly naturally selected trait, with variation due to both genetic and developmental causes^1^. An Asian swallowtail butterfly, *Papilio polytes*, shows female-limited polymorphism in Batesian mimicry^2^. The Ryukyu Islands (including Okinawa Island) are home to two female forms, forma *cyrus* and f. *polytes*. Forma *cyrus* is the non-mimetic form (Fig. 1a: upper left); it resembles males of the same species (Fig. 1a: upper right), with a white line on black hind wings. The hind wings of f. *polytes* (Fig. 1a: lower left) have a conspicuous large white spot surrounded by several small red spots, similar to the pattern of a poisonous butterfly, *Pachliopta aristolochiae* (Fig. 1a: lower right). Experimental evidence shows that birds on Miyako Island that have fed on *P*. *aristolochiae* avoid *P*. *polytes* f. *polytes*^3^. The wing colour pattern of f. *polytes* is, therefore, considered a mimic of aposematic coloration. The size of the colour spots varies widely among individuals (Fig. 1b); the size of the white spots is heritable but that of the red spots is not^4^. Recently we found that the white spot size is an important mimicry trait under strong natural selection^4^. But the ecological mechanisms causing the variation in the red spot size are unknown.

Ultraviolet irradiation (UV; 280–400 nm) poses an environmental threat to many organisms^5^. It can damage exposed tissues, and damaged wings can reduce reproductive success in butterflies^6^. Hence, flying insects such as butterflies are likely to have strategies to protect their wings against UV damage. Many organisms use melanin to protect against UV irradiation^7^. Melanin is the black pigment in the wings of *P*. *polytes*, and so is not involved in the expression of the mimetic red and white spots^8^. The conflict between protecting against UV damage and avoiding predation suggests a trade-off.

We hypothesized that under strong UV irradiation, butterflies develop a wider melanized (black) area, which results in smaller mimetic red spots. We tested the following two predictions based on this hypothesis: (1) The red spot size of f. *polytes* changes seasonally owing to seasonal fluctuation of UV radiation; and (2) UV irradiation during the larval stage of the current generation or during the adult stage of the maternal generation reduces the red spot size by an epigenetic mechanism.

## Results

### Phenotypic trade-off between black and red areas

**Figure 2:**
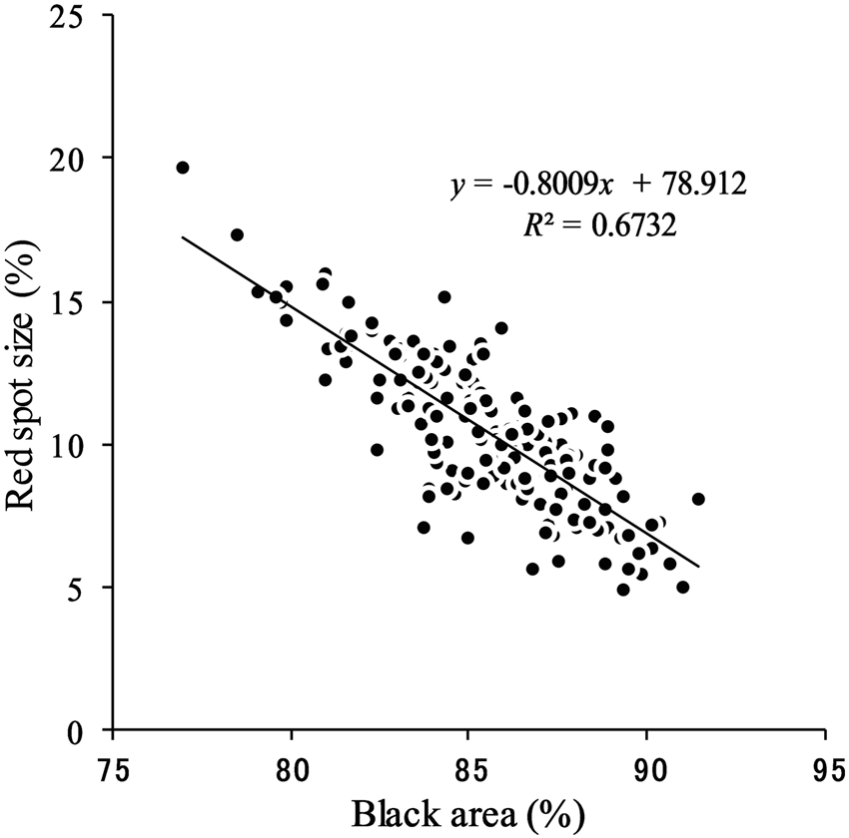
Relationship between background black area and red spot size in hind wings of *Papilio polytes* f. *polytes*. The solid line is the regression. The regression equation was obtained by least-squares linear regression analysis.

We first tested the hypothesis of a phenotypic trade-off between the red spot size and the background black area on hind wings. We estimated the correlation between those values of f. *polytes* collected on Okinawa Island in 2014 and 2015. The background black area (%) was defined as 100 × (hind-wing area − [red + white mimetic spot areas])/hind-wing area^4^. There was a significant negative correlation between them (*r* = −0.82, *P* < 0.0001, *n* = 181, Fig. 2).

### Seasonal changes in red spot size

**Figure 3:**
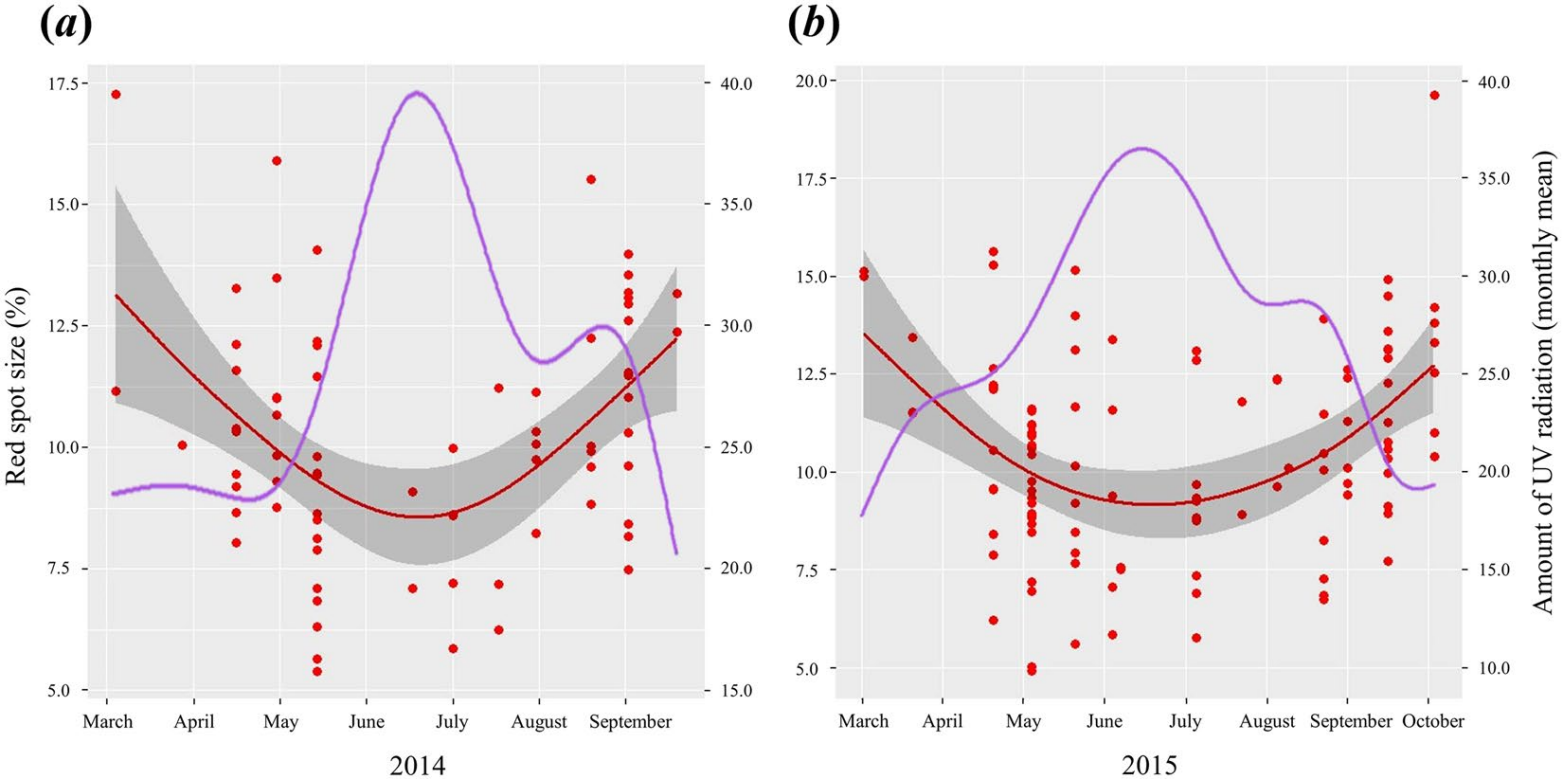
Seasonal variations in red spot size and amount of solar UV-B radiation on Okinawa in (**a**) 2014 and (**b**) 2015. The red curves indicate the smoothing spline fitted to the red spot size, and the grey bands indicate the 95% confidence intervals of the means. The purple curves are fitted to the amount of solar UV radiation.

The red spot size of females captured in 2014 and 2015 varied seasonally and was significantly negatively correlated with the cumulative intensity of the solar UV-B radiation of the month in which they were captured (2014, *P* < 0.01, *n* = 73, Fig. 3a; 2015, *P* < 0.0001, *n* = 108, Fig. 3b). The females had smaller red spots during June and July, when the solar UV-B radiation was strongest.

The red spot size of f. *polytes* (mean ± SD: 10.50 ± 2.64, *n* = 108) collected in 2015 on Okinawa was significantly smaller (*P* < 0.0001, Welch’s *t*-test) than that of its model, *P*. *aristolochiae* (13.77 ± 0.84, *n* = 10), and its variance was larger (*F* = 0.107, *df* = 9, 107, *P* < 0.01, *F*-test).

### Effect of experimental UV exposure on red spot size

**Figure 4:**
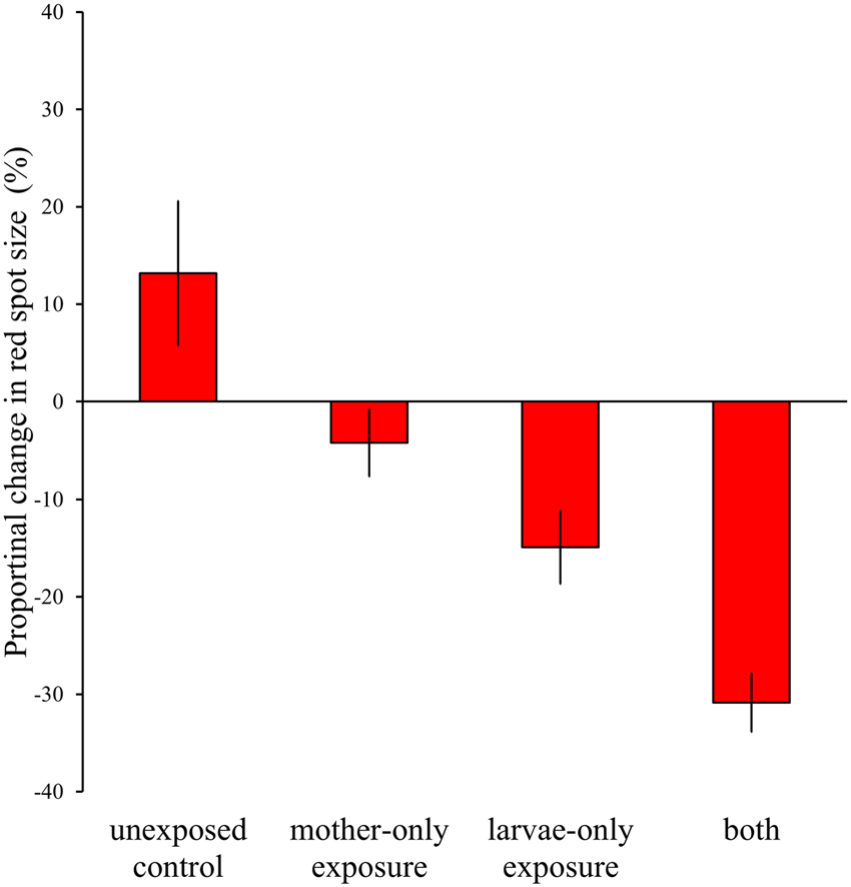
Proportional change in the red spot size. Data from two years are combined. Bars represent ± SEM. Effects of exposure at maternal stage and larval stage are independent and significant (mother-only exposure, *t* = −2.676, *P* = 0.008; larva-only exposure, *t* = −4.831, *P* < 0.0001; interaction, *t* = 0.231, *P* = 0.81, *df* = 99 in full ANOVA model; mother-only exposure, χ^2^ = 6.92, *df* = 1, *P* < 0.01; larva-only exposure, χ^2^ = 21.21, *df* = 1, *P* < 0.0001; interaction, χ^2^ = 0.04, *df* = 1, *P* = 0.82 in the likelihood test).

**Figure 5:**
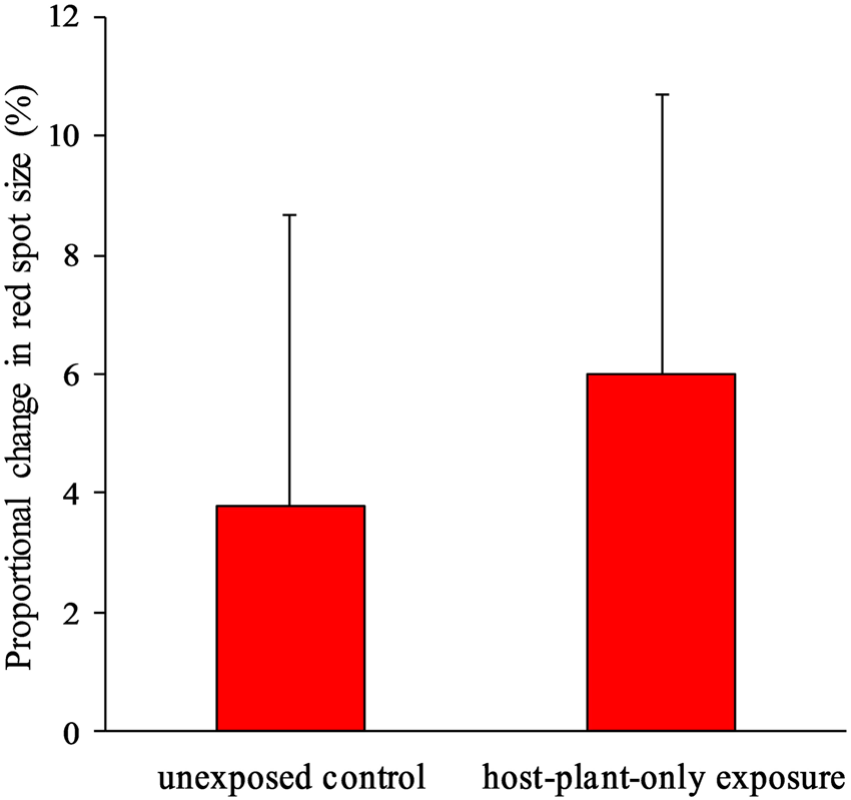
Influence of exposure of host plant leaves to UV on the change in red spot size of the butterflies. Error bars represent ± SEM.

The red spot size of females exposed to UV radiation at the larval stage was significantly smaller than that of unexposed controls (Fig. 4). Remarkably, that of females whose mothers were exposed to UV was also smaller, suggesting an epigenetic effect. Those effects were additive and independent, as the effect of their interaction was not significant in either the likelihood test or the full-model ANOVA (Fig. 4). The effect was not caused by a change in host plant quality by UV irradiation, because feeding of larvae on UV-exposed leaves did not significantly affect the red spot size (*F*_1,7_ = 0.04, *P* = 0.84, Fig. 5). We also investigated whether UV exposure in the laboratory caused not only a wider black area but also more-melanized background black areas. UV irradiation slightly reduced the average brightness value (lower brightness = darker wing), but not significantly so (*F*_1,11_ = 1.95, *P* = 0.19, nested ANOVA; Supplementary Information).

## Discussion

Our results clearly show a negative correlation between UV irradiation and red spot size in the field (Fig. 3). In the laboratory, UV irradiation at the egg and larval stages reduced the red spot size at the adult stage (Fig. 4). This was not caused by UV irradiation of the food plants (Fig. 5). Furthermore, UV irradiation of adult females reduced the size of red spots of their daughter butterflies (Fig. 4). These results support the hypothesis that mimetic females of *P*. *polytes* flexibly increase the black area of the hind wings in an environment with strong UV, which consequently reduces the red spot size.

Forma *polytes* has both the white spot and the red spot mimetic traits on the hind wings. Katoh *et al*.^4^ reported that the white spot size had heritable variation, but that the red spot size was not heritable. However, our field data and results of the UV exposure experiment indicate that the red spots can respond flexibly to environmental changes. An important finding is that maternal-experienced environments affected offspring phenotypes through putative epigenetic effects. *Papilio polytes* (as well as the model, *P*. *aristolochiae*) is multivoltine, and on Okinawa the egg-to-adult growth time is about 1 month (M. Katoh, unpublished data). Therefore, it is highly likely that larvae will be exposed to UV of a similar strength to what their mothers experienced. Given this similarity in environmental UV strengths between parent and offspring generations, the observed epigenetic effect could be a result of an adaptive strategy by which offspring can prepare to allocate more melanin to protecting wings from UV when necessary.

This result raises a new question of UV-irradiation experiment. As strong UV radiation reduces the red spot size of f. *polytes*, the putative aposematic red spot becomes more dissimilar to the model’s. Does this response therefore degrade the mimetic effect? One possibility is that the white spot of f. *polytes* is the most salient mimetic signal to predators, outweighing other imperfect mimetic traits^9^ and maintaining the mimetic efficiency. Another possibility is that the increased UV protection function compensates for the fitness cost of the increased predation risk due to imperfect mimicry. To test these ideas, we should empirically examine the effect of wing degradation by UV radiation on fitness of *P*. *polytes* in the field. It is important also to note that butterfly wing colour patterns have various functions such as thermoregulation^10,11^ and mate choice^12^, besides mimetic signals^13^.

Epigenetic effects in which parent-experienced environments affect phenotype expression of their offspring are known to have some basic mechanisms, such as DNA methylation, histone acetylation, and microRNA expression^14^. The mimetic coloration of *P*. *polytes* in the Ryukyu Islands provides a good opportunity to study the relative importance of those developmental and ecological mechanisms in insects.

## Methods

### Measurement of spot size

The red spot size is defined as the area of red spots relative to the area of the hind wing. We measured this in the following way^4^: The wing of each butterfly (ventral view) was photographed with a digital camera (Nikon D7000, Nikon Corporation, Tokyo, Japan) mounted 50 cm directly above the specimen under invariant light (Fig. S1). The left wing was used unless it was damaged. We analysed the hind-wing area below a horizontal line (Fig. S1) demarcated as follows. We defined landmark 1 as the intersection of the second anterior cubitus vein (the border between space 1b and space 2) and the border of the cell on the hind wing, and landmark 2 as the tip of the vein on the tail of the hind wing. The line connecting landmarks 1 and 2 was rotated in Adobe Photoshop CS5 (Adobe Systems Inc., San Jose, CA, USA) so that it was vertical. Then a horizontal line crossing landmark 1 was drawn perpendicular to the vertical line. The red spot size was defined as the sum of the total area of the red spot on the hind wing divided by the total hind-wing area (the area bounded by a dotted line in Fig. S1) measured in ImageJ software (National Institutes of Health, Bethesda, MD, USA).

We similarly measured the red spot sizes of the model, *P*. *aristolochiae*, for comparison.

The dorsal side of wings would be more likely to be exposed to UV light than the ventral side. However, we used the ventral side because we did not need to open the wings. There was a strong correlation in the red spot size between the dorsal and ventral sides in mimetic females of *P*. *polytes* (*r* = 0.78, *P* < 0.0001, *n* = 66, collected in the field population on Okinawa Island in 2011–2013).

### Seasonal variation in red spot size

In the daytime (09:00–14:00) at intervals of 4 to 33 days during 2014 to 2015, we captured mimetic *P*. *polytes* females in a field of Nakijin Village on Okinawa and measured their red spot sizes to reveal any seasonal pattern.

### Comparison of red spot size with the model’s

In 2015 on Okinawa, we measured the red spot sizes of the model, *P*. *aristolochiae*, captured in the field (*n* = 10) from May to October and compared it with that of f. *polytes* (*n* = 108) specimens collected in the field from March to October.

### Laboratory rearing and UV-irradiation experiment

A laboratory experiment was conducted in 2015 and 2016. We captured mimetic females in Nakijin in June of each year. As those butterflies were collected in the field, their age was unknown. Adult females were kept in paper triangles, brought to the laboratory, and randomly assigned to one of two groups the next morning (between 06:00 and 07:00). The first group was kept in an incubator (25 ± 1 °C, 13-h light/11-h dark, 70 cm × 58 cm × 102 cm high) with a UV lamp (Vivarium Glow Power UVB, 20 W, Pogona-Club, Saitama, Japan). The other (control) was kept in the same condition but with a fluorescent lamp (FL20S.D-EDL-D65, 20 W, Toshiba, Tokyo, Japan). Note that all of these butterflies were always kept individually in paper triangles to avoid damage due to flying in the incubator (except at feeding time). The paper triangles block only about 9.3% of UV radiation (M. Katoh, unpublished data). The air temperature in the incubators was automatically controlled. We confirmed that the difference in lamps did not affect the temperature inside the incubators (M. Katoh unpublished data). Once a day we fed the females with 15% sucrose solution until they stopped feeding. The butterflies in the UV group were exposed to UV from 06:00 to 19:00 for 3 days (except at feeding time). We let the butterflies in which both groups oviposit on fresh leaves of *Toddalia asiatica* in the laboratory under fluorescent light (25 ± 1 °C, 13-h light/11-h dark) on days 4 and 5. After oviposition, the eggs were immediately transferred to one of the two randomly assigned incubators (UV or control). Hatched larvae were reared in those incubators on fresh *T*. *asiatica* leaves. As the final butterfly wing colour patterns are determined during late larval to pupal development^15–17^, we timed the UV exposure to fall before this stage. Gut-purged larvae were moved to the laboratory for pupation. The emerged butterflies were killed within 4 h after emergence and stored in a freezer at −30 °C to avoid physical damage to their wings. We assigned 21 larvae from 8 mothers to the unexposed control, 19 larvae from 6 mothers to the mother-only exposure treatment, 33 larvae from 10 mothers to the larva-only exposure treatment, and 32 larvae from 7 mothers to the both-exposure treatment. Note that although we use the term “larval exposure” for simplicity, we exposed both egg and larval stages.

We calculated the standardized red spot size of the females in the two generations as 100× (red spot size of the lab-bred female − red spot size of its field-collected mother)/(red spot size of the field-collected mother) under the four exposure treatments.

As both butterflies and host plant leaves were held under UV, a change in host plant quality caused by UV exposure might have indirectly caused a change in the red spot size. To test this possibility, we exposed leaves of *T*. *asiatica* in the incubator to UV as in the main experiment for 1 to 2 days and then provided them to the larvae of mother butterflies collected in Nakijin Village in 2016 and 2017. We assigned 23 larvae from 4 mothers to the host-plant-only exposure treatment and 14 larvae from 5 mothers to the unexposed control.

### Statistical analysis

All analyses were performed in R v. 3.2.3 software^18^. We used a generalized additive model (mgcv in R) to test the relationship between the seasonal variation in the red spot size and UV intensity in the field. The red spot size of field-collected mimetic females was the response variable, and the average daily integrated solar UV-B radiation on Okinawa in the month when each female was collected (obtained from the Japan Meteorological Agency) was the explanatory variable. Smoothing splines were fitted to each data set. We tested the influence of UV irradiation on the expression of the red spot size using a generalized linear mixed model (GLMM, lme4 in R) with a Gaussian error structure and an identity link function, using maternal exposure, larval exposure, and their interaction as fixed effects and year as a random effect. We assessed the significance of each effect using the likelihood-ratio test (ANOVA of the model with a particular explanatory variable vs the model without that variable) and ordinal ANOVA of the full model. To test any effect of a change in host plant quality, we used a GLMM with treatment as the fixed factor and mother as the random factor.

## Electronic supplementary material

Supplementary Information

## References

[1] Nijhout HF, Cinderella M, Grunert LW (2014). The development of wing shape in Lepidoptera: mitotic density, not orientation, is the primary determinant of shape. Evol. Dev..

[2] Uesugi K (1996). The adaptive significance of Batesian mimicry in the swallowtail butterfly, *Papio polytes* (Insecta, Papilionidae): Associative learning in a predator. Ethology.

[3] Clarke CA, Sheppard PM (1972). The genetics of the mimetic butterfly *Papilio polytes* L. Phil. Trans. R. Soc. Lond. B.

[4] Katoh, M., Tatsuta, H. & Tsuji, K. Rapid evolution of a Batesian mimicry trait in a butterfly responding to arrival of a new model. *Sci*. *Rep*. **7**, 6369, https://www.nature.com/articles/s41598-017-06376-9 (2017). 10.1038/s41598-017-06376-9 PMC5527021 28743998

[5] Hansson LA, Hylander S (2009). Size-structured risk assessments govern *Daphnia* migration. Proc. R. Soc. Biol. Sci. Ser. B.

[6] Fischer K, Perlick J, Galetz T (2008). Residual reproductive value and male mating success: older males do better. Proc. R. Soc. B.

[7] Zellmer ID (1995). UV-B-tolerance of alpine and arctic *Daphnia*. Hydrobiologia.

[8] Nishikawa, H. *et al*. Molecular basis of wing coloration in a Batesian mimic butterfly, *Papilio polytes*. *Sci*. *Rep*. **3**, 3184, https://www.nature.com/articles/srep03184 (2013). 10.1038/srep03184 PMC3822385 24212474

[9] Kazemi B, Gamberale-Stille G, Tullberg BS, Leimar O (2014). Stimulus salience as an explanation for imperfect mimicry. Curr. Biol..

[10] Watt WB (1968). Adaptive significance of pigment polymorphisms in *Colias* butterflies. I. Variation of melanin pigment in relation to thermoregulation. Evolution.

[11] Watt WB (1969). Adaptive significance of pigment polymorphisms in *Colias* butterflies. II. Thermoregulation and photoperiodically controlled melanin variation in *Colias eurytheme*. Proc. Natl. Acad. Sci. USA.

[12] Jiggins CD, Naisbit RE, Coe RL, Mallet J (2001). Reproductive isolation caused by colour pattern mimicry. Nature.

[13] Brakefield PM, Reitsma N (1991). Phenotypic plasticity, seasonal climate and the population biology of *Bicyclus* butterflies (Satyridae) in Malawi. Ecol. Entomol..

[14] Mukherjee K, Twyman RM, Vilcinskas A (2015). Insects as models to study the epigenetic basis of disease. Prog. Biophys. Mol. Biol..

[15] Nijhout HF (1980). Pattern formation on lepidopteran wings: Determination of an eyespot. Dev. Biol..

[16] Yoshida A, Aoki K (1989). Scale arrangement pattern in a lepidopteran wing. 1. Periodic cellular pattern in the pupal wing of *Pieris rapae*. Dev. Growth Diff..

[17] Koch PB (2000). Insect pigmentation: activities of beta-alanyldopamine synthase in wing color patterns of wild-type and melanic mutant swallowtail butterfly *Papilio glaucus*. Pigment Cell Res..

[18] R Core Team. R: A language and environment for statistical computing. R Foundation for Statistical Computing, Vienna, Austria, https://www.r-project.org/ (2015).

