# Supplementary material for: Ultraviolet exposure has an epigenetic effect on a Batesian mimetic trait in the butterfly Papilio polytes
Source: Sci Rep. 2018 Sep 7;8:13416. doi: 10.1038/s41598-018-31732-8 (PMC6128867; doi:10.1038/s41598-018-31732-8)
Supplement: Supplementary file 1 — Supplementary Information [file 41598_2018_31732_MOESM1_ESM.doc]

*Scientific Reports*

# Ultraviolet exposure has an epigenetic effect on a Batesian mimetic trait in the butterfly *Papilio polytes*

Mitsuho Katoh^1,2^*, Haruki Tatsuta^1,2^, and Kazuki Tsuji^1,2^*

^1^ Department of Agro-Environmental Sciences, Faculty of Agriculture, University of the Ryukyus, Okinawa 903-0213, Japan

^2^ The United Graduate School of Agricultural Sciences, Kagoshima University, Korimoto 1-21-24, Kagoshima 890-8580, Japan

*Correspondence to: ryukyu523@gmail.com (M.K.); tsujik@agr.u-ryukyu.ac.jp (K.T.)

**Supplementary Information**

**Image trimming of specimen**

The hind-wing area bounded by a dotted line was used for the analysis (Fig. S1). This area was defined as shown in Fig. S1 (see the main text for details).

**Measurement of specimens with ambiguous colour spot areas**

In 171 of 181 specimens collected in 2014 and 2015^1^, the white spot was distinct from the background black area and from the red areas. In the other 10 specimens, however, the distinction was unclear, because adjoining white and red spots were partially fused. In such cases, we pseudo-randomly chose 30 points (pixels) from the clearly white spot area (Fig. S2: the area enclosed by black circles) with the ‘multi-point’ tool of ImageJ software, converted the image to grey scale, and evaluated the brightness value (0–255) of each pixel. We defined a pixel as being included in the white spot area when its brightness value was higher than the mean − SD brightness value of the focal butterfly. We specified such white areas using the ‘threshold’ tool (Fig. S3), then measured the red spot area by excluding the white spot area (Fig. S3a) from the non-black area (Fig. S3b). When this procedure was tested on specimens with distinct red spots, the number of pixels judged as the red spot area was almost exactly the same as that in the spots identified by eye.

**Effect of UV irradiation on the darkness of black area**

To investigate whether UV irradiation promotes not only wider but also darker (more-melanized) background black areas, we measured the average brightness values (lower brightness = darker wing) of the hind-wing of f. *polytes* females that emerged in the UV exposure experiment in ImageJ software. We used butterflies of the unexposed control treatment (*N* = 21) and of the both-exposure treatment (*N* = 32). A generalized linear mixed model, with treatment (UV irradiation vs control) nested within the effect of year as the fixed effect and mother as the random factor, indicated that UV irradiation did not significantly reduce the brightness value (mean ± SD: unexposed control = 36.92 ± 5.20, both-exposure treatment *=* 35.32 ± 3.96, *F*_1,11_ = 1.95, *P* = 0.19).

**Reference**

1. Katoh, M., Tatsuta, H. & Tsuji, K. Rapid evolution of a Batesian mimicry trait in a butterfly responding to arrival of a new model. *Sci. Rep*. **7**, 6369; 10.1038/s41598-017-06376-9 (2017).

Figure S1. Delineation of the hind-wing. The specimen was collected by the author.


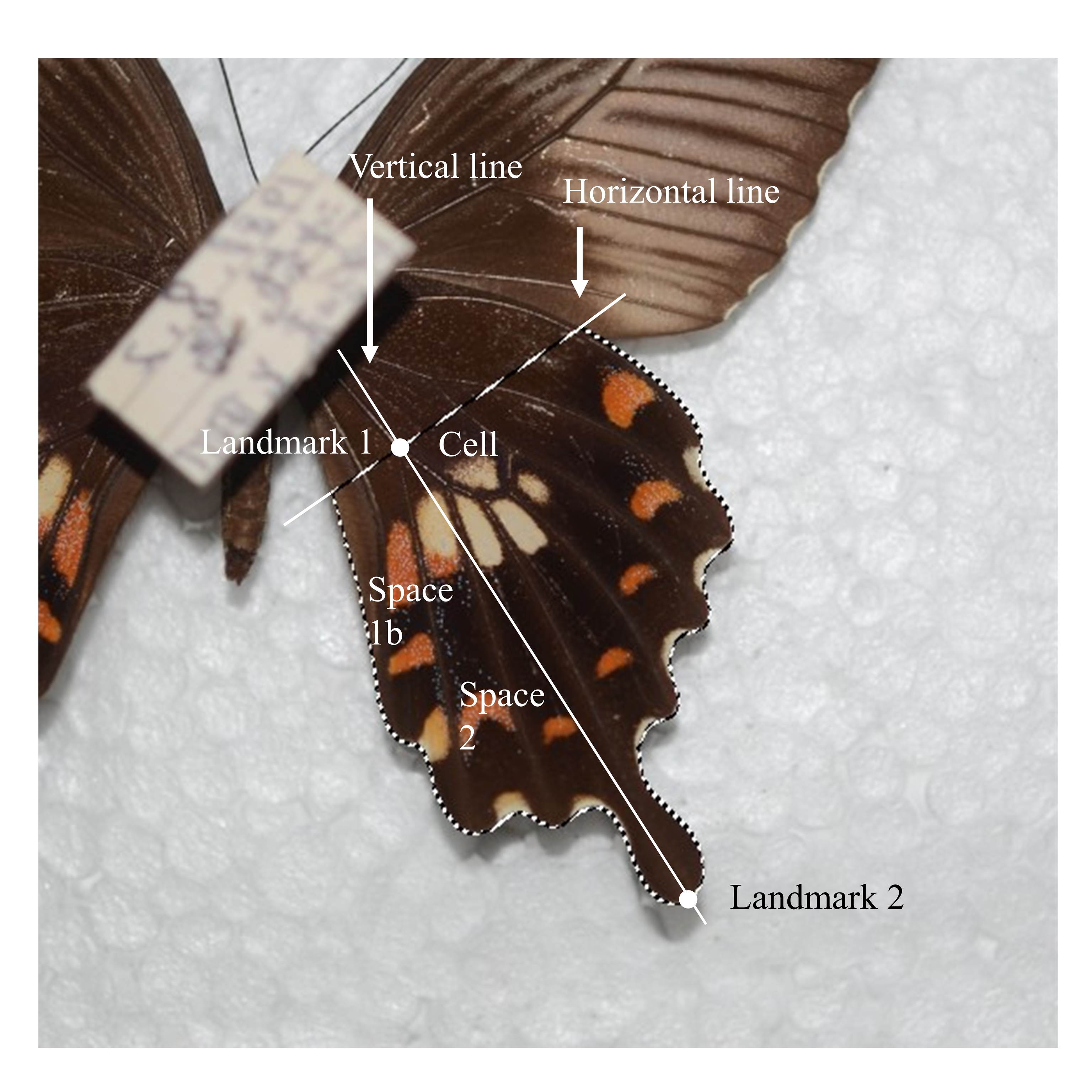


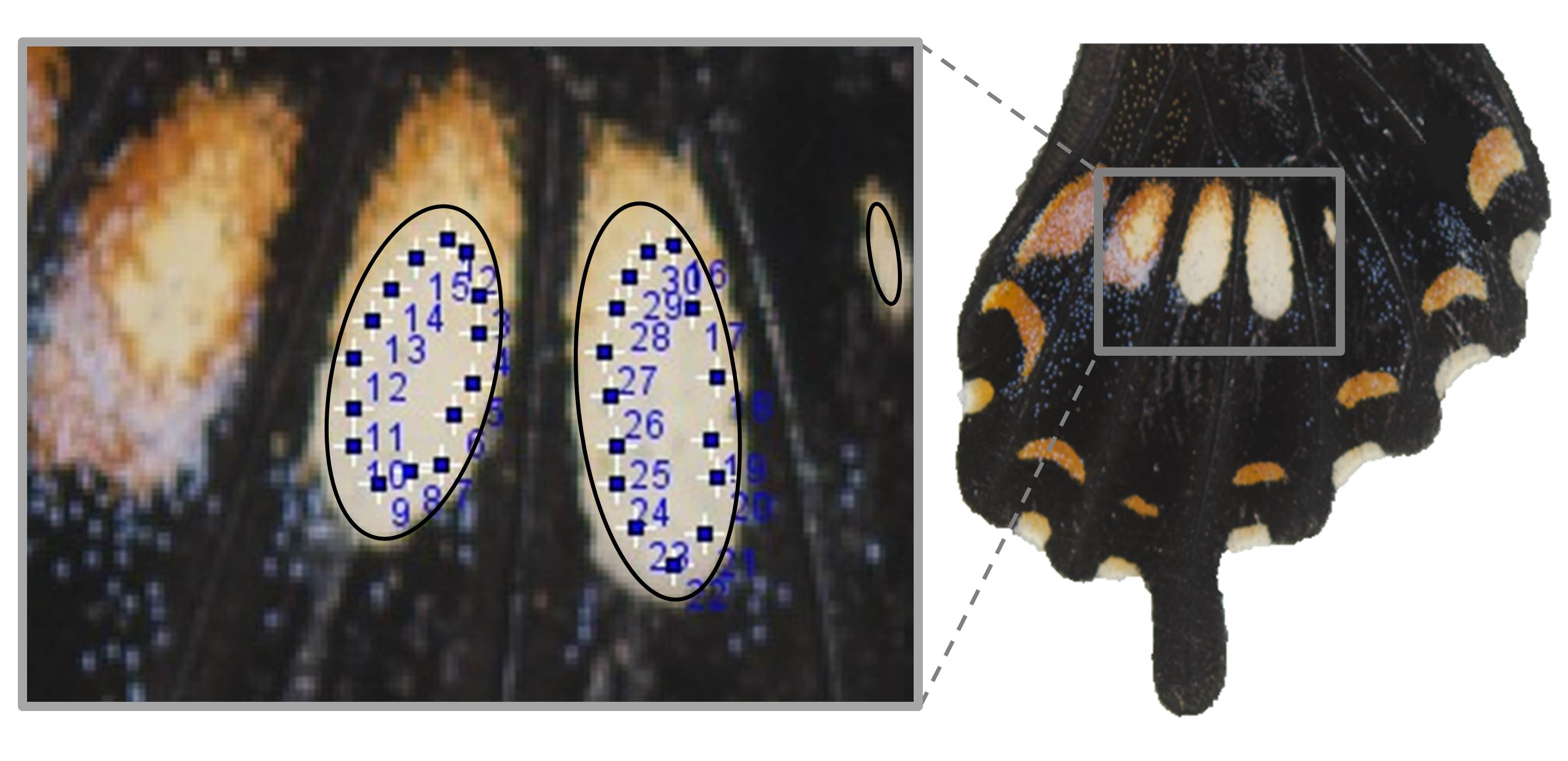
Figure S2. Thirty points were chosen from the clearly white spot area enclosed by black ellipses and were numbered in the order shown.


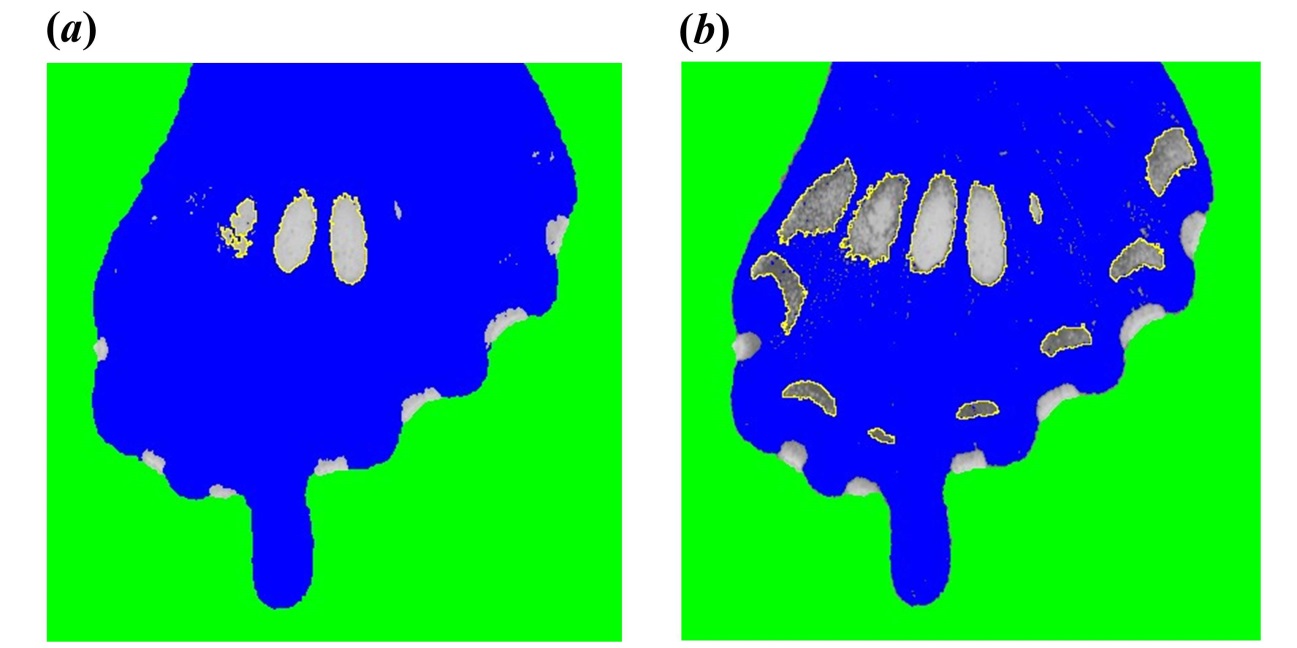
Figure S3. (*a*) White spot area; (*b*) non-black area. Green: image background; blue, hind-wing background.
